# Anhedonia is associated with computational impairments in reward and effort learning in young people with depression symptoms

**DOI:** 10.1017/S0033291725102523

**Published:** 2025-11-17

**Authors:** Angad Sahni, Anna-Lena Frey, Ciara McCabe

**Affiliations:** School of Psychology and Clinical Language Sciences, University of Reading, Reading, UK

**Keywords:** adolescent, anhedonia, depression, effort, learning, reward, youth

## Abstract

**Background:**

Anhedonia and depression symptoms have been linked to potential deficits in reward learning. However, how anhedonia impacts the ability to adjust and learn about the effort required to obtain rewards remains unclear.

**Methods:**

We examined young people (*N* = 155, 16–25 years) with a range of depression and anhedonia symptoms using a probabilistic instrumental reward and effort learning task. Participants were asked to learn which options to choose to maximize reward or minimize effort for reward. We compared the exerted effort (button pressing speed) for high (puppy images) vs low (dog images) rewards and collected subjective reports of “liking,” “wanting,” and “willingness to exert effort.” Computational models were fit to the learning data and estimated parameter values were correlated with depression and anhedonia symptoms.

**Results:**

As depression symptoms and consummatory anhedonia increased, reward liking decreased, and as anticipatory anhedonia increased, liking, wanting, and willingness to exert effort for reward decreased.

Participants exerted more effort for high rewards than for low rewards, but anticipatory anhedonia diminished this difference.

Higher consummatory anhedonia was associated with poorer reward and effort learning, and with increased temperature parameter values for both learning types, indicating a higher tendency to make exploratory choices. Higher depression symptoms were associated with lower reward learning accuracy.

**Conclusion:**

We provide novel evidence that anhedonia is associated with difficulties in modulating effort as a function of reward value and with the underexploitation of low effort and high reward options. We suggest that addressing these impairments could be a novel target for intervention in anhedonic young people.

## Introduction

Depression is the leading cause of illness and disability worldwide (World Health Organization, 2017). Anhedonia, a lack of interest and pleasure, is a core symptom of depression (American Psychiatric Association, 2013) and is characterized by blunted liking and wanting of rewards in adults (Argyropoulos & Nutt, 2013; Kaya & McCabe, 2019; Rizvi, Pizzagalli, Sproule, & Kennedy, 2016; Treadway & Zald, 2011) and young people (Ely et al., 2021; Forbes & Dahl, 2012; Kaya & McCabe, 2019; Ma, Sahni, & McCabe, 2024; McCabe, 2018). Deficits in reward learning have also been observed in depression (Kumar et al., 2018; Thomsen, 2015) and have been linked to anhedonia (Kangas, Der-Avakian, & Pizzagalli, 2022). However, it remains unclear how learning about the effort required to attain rewards may be associated with depression or anhedonia symptoms.

Depressed individuals fail to exert more effort for higher or more likely rewards (Horne, Topp, & Quigley, 2021), which suggests poor effort modulation as a function of reward. Anhedonia has been shown to be associated with lower physical effort exertion and willingness to expend effort (making low effort/low reward choices) for monetary rewards in both adults (Cléry-Melin et al., 2011; Darrow et al., 2023; Geaney, Treadway, & Smillie, 2015; Tran, Hagen, Hollenstein, & Bowie, 2021; Treadway, Bossaller, Shelton, & Zald, 2012; Yang et al., 2014) and young people (Bryant, Winer, Salem, & Nadorff, 2017; Olino et al., 2021; Slaney et al., 2023; Treadway et al., 2009). We have extended these findings by showing that anhedonia in young people is also associated with less physical effort exertion (button presses) for primary rewards, such as chocolate (Rzepa & McCabe, 2019), and less subjective willingness to exert effort (rated on a visual analogue scale) for puppy images (Frey et al., 2023).

Although few studies have examined effort learning in depression, a recent study using learning tasks with reward and punishment outcomes, as well as effort and delay costs, found reduced physical and cognitive effort exertion for monetary reward in depression (Vinckier et al., 2022). Further, utilizing computational modelling, Vinckier et al. found that, compared to controls, depressed individuals demonstrated a higher sensitivity to effort cost, which was measured as the mean aversive value of effort items in preference tasks and as the weight of effort cost on net expected value in performance tasks. However, when examining participants’ ability to update choices to maximize monetary gain (reward learning) and minimize monetary loss (punishment learning), no significant correlation was found with anhedonia symptoms, which the authors suggest could be due to the small sample size.

To extend this past work, the aim of the present study was to combine our recently adapted probabilistic instrumental reward and effort learning task (Frey et al., 2023) with a computational modelling approach. Our task is based on a probabilistic learning task from Skvortsova et al. (2017) and Skvortsova, Palminteri, and Pessiglione, 2014), which we previously adapted from utilizing monetary outcomes to including primary rewards (chocolate and puppy images). As part of the task, participants are shown shape pairs and asked to learn which shape to choose to maximize their reward outcomes and to minimize the physical effort required to obtain the rewards. Using this task, we previously found that higher anticipatory anhedonia was significantly associated with lower reward learning accuracy (Frey et al., 2023). However, we did not observe a significant association between depression or anhedonia symptoms and effort learning, which may have been the case because the task was too challenging with an interleaved design. Specifically, participants needed to switch between learning about reward and effort from trial to trial, which may have led to a trade-off between effort and reward learning. As the primary rewards used in the task were particularly salient, participants’ attention may have been shifted towards learning from rewards, rather than from effort outcomes, resulting in a ‘floor effect’ of poor effort learning across all participants, independent of anhedonia symptoms. To address this issue, the current study further adapted the task into a block design that separates reward and effort learning.

Using computational modeling, we aimed to examine the relation between depression and anhedonia and parameter values that capture different aspects of learning. Several previous studies have reported that depression and anhedonia symptoms are associated with lower reward sensitivity parameters (Chen et al., 2015; Huys, Pizzagalli, Bogdan, & Dayan, 2013; Katz, Matanky, Aviram, & Yovel, 2020). However, findings regarding reward learning rates, the weight given to unexpected outcomes that modulate future actions, have been inconsistent. Some studies find higher (Beevers et al., 2013) and others lower (Chen et al., 2015; Cooper et al., 2014; Frey, Frank, & McCabe, 2021) learning rates in depression, compared to controls. In line with these inconsistencies, a recent meta-analysis examining reinforcement learning parameters from decision-making tasks with reward and punishment outcomes found no differences in learning rates between patients with depression or anxiety and controls (Pike & Robinson, 2022). However, this may partly have been the case because the studies included in the meta-analysis differed in terms of the tasks and computational models used. To account for this, the authors employed a novel meta-analytic method to estimate the learning rates. Using this simulated approach, higher punishment learning rates and slightly lower reward learning rates were found in patients compared to control individuals (Pike & Robinson, 2022).

Another parameter that is often examined is temperature, which governs the extent to which individuals exploit high-valued actions or explore lower-valued alternatives, with higher temperature parameter values being associated with more random/exploratory choices. Some studies find that depressed individuals make more random/exploratory choices (Kunisato et al., 2012; Rupprechter et al., 2018). Using a conventional meta-analysis design, Pike and Robinson (2022) found lower *inverse* temperature (indicating more exploratory/random choices) in patients, compared to controls, but this effect was not apparent in the simulated meta-analysis.

Beyond examining depression in general, some studies have shown that individuals with higher anhedonia levels, in particular, demonstrate lower reward learning rates (Brown et al., 2021; Chase et al., 2010) and lower reward sensitivity (Huys et al., 2013) compared to those with lower anhedonia symptoms. However, few studies have investigated the dimensional relationship between anhedonia and reward learning parameters. Moreover, we are not aware of any studies exploring the relationship between effort learning parameters and depression or anhedonia symptoms. Therefore, the current study aimed to specifically examine the relation between depression and anhedonia symptoms and effort, as well as reward, learning accuracy, and parameter values. Based on previous studies, we hypothesized that higher depression and anhedonia symptoms would be associated with lower reward learning rates and higher temperature parameter values. Given the increased sensitivity to effort cost reported in depression (Vinckier et al., 2022), we expected to observe higher effort learning rates with increasing depression symptoms. In addition, in line with our and others’ findings, we hypothesized that young people with higher levels of depression and anhedonia would show lower subjective liking, wanting and willingness to exert effort for rewards.

## Methods

### Participants

Using G*Power we calculated that a sample size of at least 84 participants was required to examine correlations between task measures and symptoms, with a medium effect size of 0.3, 80% power and *α* = 0.05.

Young people (*N* = 155) between the ages of 16 and 25 years, with a range of depression symptoms, were recruited from local schools and the university student population via the School of Psychology research panel, online advertisements, and posters displayed throughout the university.

The study was approved by the School of Psychology Research Ethics committee (ref no: 2023–150-CM) and complies with the Helsinki Declaration of 1975, as revised in 2013. After reading the information sheets, all participants provided informed consent.

University participants were reimbursed for their time with course credits or were entered into a draw for a £20 Amazon voucher. All participants received a debrief sheet, which advised those concerned about their mood to contact their doctor and provided contact details for the Samaritans.

### Questionnaires

Participants filled out a demographics form and the below questionnaires online.

The Beck Depression Inventory-II (BDI; Beck et al., 1961) is a widely used depression measure consisting of 21 multiple-choice items. Each item has four response options, corresponding to a score of 0 to 3. The total score, summed across all items, is interpreted as follows: 0–13 = low/no depression, 14–28 = mild/moderate depression, and 29–63 = severe depression.

The Temporal Experience of Pleasure Scale (Gard, Gard, Kring, & John, 2006) is an anhedonia measure consisting of 18 items, each rated on a 6-point Likert scale from “very false for me” to “very true for me.” The TEPS comprises two subscales measuring anticipatory pleasure (TEPS-A) and consummatory pleasure (TEPS-C), respectively. Lower scores on the TEPS indicate a greater severity of anhedonia.

The Snaith–Hamilton Pleasure Scale (SHAPS; Snaith et al., 1995) is a 14-item questionnaire used to assess anhedonia. Each item is scored as either 0 or 1, with “disagree” responses (either “disagree” or “strongly disagree”) receiving a score of 1, and “agree” responses (either “agree” or “strongly agree”) receiving a score of 0. The total score ranges from 0 to 14, with higher scores indicating a greater degree of anhedonia.

The State Trait Anxiety Inventory (STAI) is an anxiety measure consisting of a state anxiety and a trait anxiety scale. The current study used the trait anxiety scale, which contains 20 items scored on a 4-point Likert scale. The sum score ranges from 20 to 80, and a total score over 60 suggests severe anxiety (Bieling, Antony, & Swinson, 1998).

Good internal consistency, test–retest reliability, and convergent and discriminant validity have been shown for the BDI (Beck et al., 1961), TEPS anticipatory and consummatory subscales (Gard et al., 2006), the SHAPS (Nakonezny et al., 2010) and STAI (Bieling et al., 1998).

After completing the questionnaire measures, participants were sent a link to the online learning task described below, which was completed on a desktop PC or laptop.

### Learning task

We adapted a probabilistic instrumental reward and effort learning task from Skvortsova et al. (2014), substituting monetary reward for images of puppies and dogs as high and low rewards, respectively. We found in our previous study that young people regard puppy images as rewarding, and as more rewarding than images of dogs (Frey et al., 2023), in line with past reports that baby animals are consistently rated as more pleasant than adult animals (Lehmann, Huis in‘t Veld, & Vingerhoets, 2013).

We simplified the task by separating the reward and effort learning trials into two separate blocks, as we (Frey et al., 2023) and others (Skvortsova et al., 2014) have observed that participants find it difficult to simultaneously learn about reward and effort in an interleaved design.

Before the task, subjects were asked to rate the reward stimuli on a visual analogue scale ranging from 0 to 100. Specifically, they were asked to indicate how much they *liked* looking at the puppy images, how much they *wanted* to see the puppy images, and how much *effort they were willing to exert* to look at the puppy images. These ratings were collected again at the end of the experiment.

During the task, each trial started with a choice between two shapes, where pressing the ‘C’ key selected the left shape and pressing the ‘M’ key selected the right shape. In the reward learning block, one shape was associated with a high reward (puppy image) 75% of the time and a low reward (dog image) 25% of the time, while the other shape was associated with a high reward (puppy image) 25% of the time and a low reward (dog image) 75% of the time. The effort level was fixed (high effort/60 button presses 100% of the time) for both shapes. In the effort learning block, one shape was associated with high effort (60 button presses) 75% of the time and low effort (35 button presses) 25% of the time, while the other shape was associated with high effort (60 button presses) 25% of the time and low effort (35 button presses) 75% of the time. The reward was fixed (high reward/puppy images 100% of the time) for both shapes. The order of the blocks and the shape pairs were randomized per participant and which side (left or right) was associated with the higher contingency was counterbalanced between blocks. Participants were instructed to choose the shapes that resulted in receiving high rewards in the reward block and in low effort requirements in the effort block.

**Figure 1.**
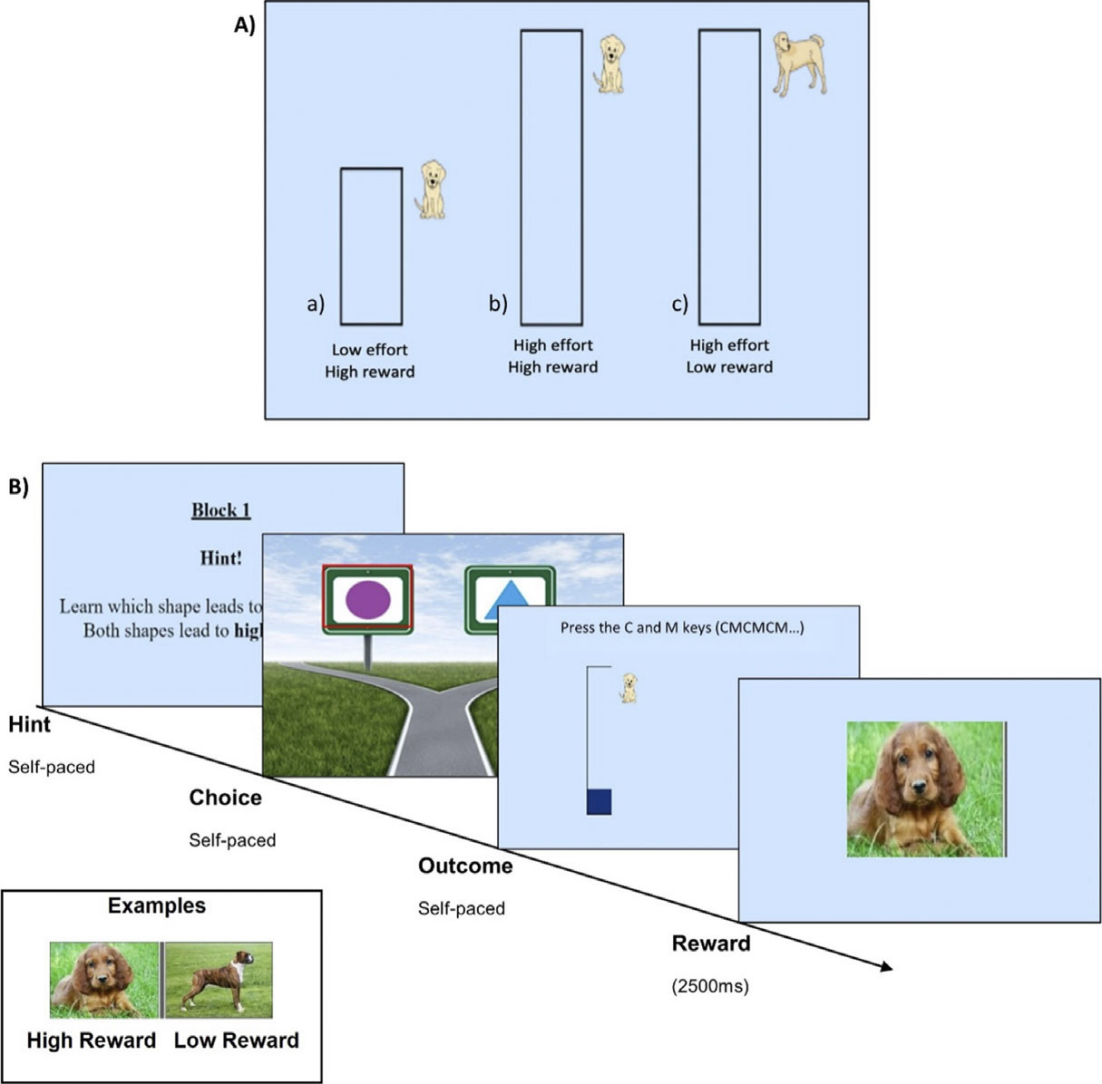
Task structure. (a) possible outcomes associated with choices, and (b) procedure of each trial, and the hint given at the beginning of each block. A 30-second break separated the reward and effort learning blocks. For ‘self-paced’ phases, the participant’s action (i.e. making a choice or filling up the effort rectangle) determined when the task moved on to the next stage.

Once a choice was made, participants were informed about the outcome, i.e. the reward and effort levels were shown on the screen (high reward: puppy line drawing, low reward: dog line drawing; low effort: a small rectangular bar to fill up, high effort: a larger rectangular bar to fill up; see Figure 1). Next, participants needed to exert effort to obtain the reward by alternatively pressing the ‘C’ and ‘M’ keys, which led to a blue bar filling up the rectangle. On high effort trials, filling up the rectangle required 60 button presses, while on low effort trials only 35 button presses were needed. After participants reached the effort target, they received the actual reward (seeing a photograph of a puppy or dog).

Overall, the task consisted of 4 practice trials followed by 50 experimental trials (block 1 with 25 trials, 30 s break, block 2 with 25 trials) and took ~30 minutes to complete. Based on previous research using similar tasks, 25 trials seemed sufficient to allow participants to learn the contingencies in the simplified block design without making the tasks so long as to cause fatigue.

The data generated consisted of the task measures of reward and effort learning accuracy, and speed of effort key presses for high and low rewards, as well as the subjective reward ratings.

### Analysis

All data were examined using R (version 4.3.2). To check for habituation effects, we used a repeated-measures ANOVA with rating type (liking, wanting, willingness to exert effort) and time (pre- or post-task) as within-subject factors and the difference in ratings (i.e. ratings for high rewards minus low rewards) as the dependent variable.

We conducted correlations between depression and anhedonia symptoms, subjective reward ratings, and task measures. When examining anhedonia, we controlled for depression, with the following 4 anhedonia items removed from the BDI: loss of pleasure, loss of interest, loss of energy, and interest in sex (Winer et al., 2014). We also examined the relationship between anxiety symptoms (controlling for BDI (full scale)) and subjective reward ratings and task measures, to assess if the observed relationships are unique to anhedonia and depression symptoms.

To assess whether task measures were more strongly associated with anhedonia than with depression symptoms, we compared the coefficients of significant correlations involving these symptoms using Pearson and Filon’s *z* method (Pearson & Filon, 1898) for comparing two dependent correlations that have one variable in common. As higher BDI scores reflect *higher* depression, while higher TEPS scores indicate *lower* anhedonia, we reverse coded the TEPS subscales before applying the calculation in order to compare effect sizes.

We further assessed if accuracies differed between the effort and reward learning blocks, while controlling for block order, by using a repeated-measures ANOVA with the order of the blocks as the between-subject factor and block type (effort or reward) as the within-subject factor.

In addition, we examined participants’ ability to modulate effort exertion as a function of reward, by calculating the average speed of button presses (number of presses per second) for the high reward/high effort trials and for the low reward/high effort trials across the whole task. We took the *difference* (effort speed for high rewards minus effort speed for low rewards) to be a measure of effort modulation, such that if the difference is positive, it indicates that participants exerted more effort (in terms of speed) for high rewards than for low rewards.

Visual inspection, using box-and-whisker plots, revealed several clear outliers in the difference between effort exertion for high and low rewards. Ten outliers that were +/− 2 SDs or more from the mean were removed from further analysis. Then, to determine if participants exerted more effort for high than for low rewards, as expected, we conducted a one-tailed, one-sample *t*-test comparing the distribution of effort differences to *μ* = 0 (representing no difference in effort exertion for high and low rewards). Additionally, to examine whether the ability to modulate effort based on reward value is associated with symptoms, we performed partial correlation analyses between the effort speed difference and depression (controlling for block order) and anhedonia (controlling for depression scores with anhedonia items removed and block order).

As most variables violated the assumption of normality, we used Spearman’s method for all correlations. Analyses were corrected for multiple comparisons by applying the Benjamini-Hochberg (BH) method (Benjamini & Hochberg, 1995).

### Computational modeling

Several Q-learning models were fit separately to the reward and effort learning data. The models contained between two and four parameters for each block, including learning rate (*α*), outcome sensitivity (*ρ*), temperature (*τ*), choice bias (*φ*, i.e. repeated item selection *independent of the outcome*), and choice bias decay (*γ*) parameters. All models contained learning rates and temperature parameters with different combinations of the other parameters (see Supplementary Materials for details). We fit two versions of each model: one that only accounted for factual learning, and another that additionally integrated counterfactual learning.

Maximum likelihood estimation was used for model fitting, and models were compared using Akaike’s Information Criterion weights (Wagenmakers & Farrell, 2004). For the best-fitting model, data simulations were performed using the estimated participant parameters, parameter recovery was examined, and the simulated and actual data were compared for model validation (see Supplement for details).

Parameter values from the best-fitting model were correlated with depression scores and with anhedonia scores, controlling for depression (with anhedonia items removed, as described above). Spearman’s method was used as the assumptions of normality were violated.

## Results

### Demographics and questionnaire measures

Table 1 describes subjects’ demographics. Participants had a mean age of 19 years and a broad range of depression and anhedonia symptoms. The sample contained participants with low (BDI 0–13, *N* = 72), mild/moderate (BDI 14–28, *N* = 65), and severe depression (BDI 29–63, *N* = 18).

**Table 1.** Demographics and symptoms of the sample.

Characteristics	Mean (SD) or frequency
	*N* = 155.
Age (years)	19.10 (1.98)
Gender split (F/M/O)	118/32/5
Ethnicities (*n*)	
White	97
BAME	48
Other	10
Reward learning accuracy (%)	68.93 (16.87)
Effort learning accuracy (%)	70.71 (16.33)
Clinical diagnosis of depression (Y/N/D)	16/129/10
BDI	15.9 (0.04)
Depression symptom severity (L/M/S)	72/65/18
SHAPS	2.19 (2.57)
TEPS total	73.81 (10.01)
TEPS-A	41.1 (7.04)
TEPS-C	32.71 (4.76)
STAI	51.81 (11.17)

Abbreviations: BAME, Black, Asian and Minority Ethnic; BDI, Beck Depression Inventory; Symptom severity, L = Low (BDI ≤ 13), M= Mild/Moderate (14 ≤ BDI ≤ 28), S= Severe (BDI ≥ 29); TEPS-A, Temporal experience of pleasure scale – anticipatory subscale; TEPS-C, Temporal experience of pleasure scale – consummatory subscale; SHAPS, Snaith-Hamilton Pleasure Scale; STAI, State Trait Anxiety Inventory; Clinical Diagnosis of Depression (previously provided by a psychiatrist/clinician), Y = Yes, N = No, D = Don’t Know.

### Symptoms and subjective ratings

To assess potential habituation to the rewards, we used a repeated-measures ANOVA and found no significant effects of rating (liking, wanting, effort willingness; *F*(2, 924) = 1.02, *p* = .361) or time (pre or post task; *F*(1, 924) = 0.05, *p* = .829), and no significant interaction (*F*(2, 924) = 0.51, *p* = .601).

**Figure 2.**
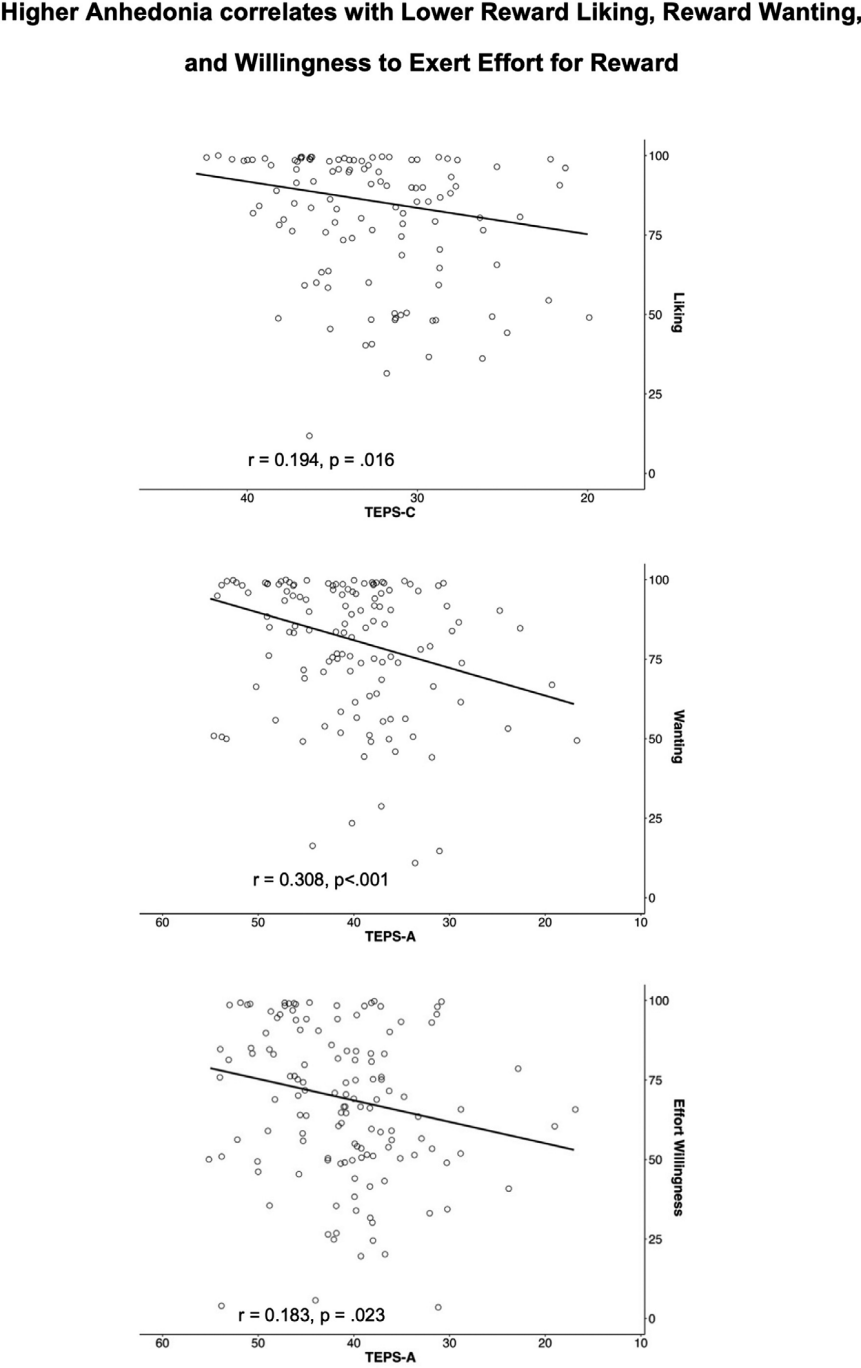
Subjective ratings plotted against anhedonia scores (higher TEPS = lower anhedonia).

In addition, we examined the relationships between symptoms and subjective ratings of puppy images at the beginning of the task. We found that, as depression symptoms (higher BDI; *r* = −0.194, *p* = .016) and consummatory anhedonia (lower TEPS-C; *r* = 0.194, *p* = .016) increased, liking of puppy images decreased. Further, as anticipatory anhedonia (TEPS-A) increased, liking (*r* = 0.308, *p* < 0.001), wanting (*r* = 0.308, *p* < 0.001) and willingness to exert effort (*r* = 0.183, *p* = 0.023) for puppy images decreased (Figure 2). After applying the BH method for multiple comparisons, the correlations for liking and wanting ratings remained significant (Supplementary Tables S1 and S2).

The only measure that was significantly associated with both depression and consummatory anhedonia was the liking rating. To examine whether liking was more strongly associated with anhedonia than with depression, we compared the correlation coefficients for these two associations using the Pearson and Filon’s *z* method. We found no significant difference (*z* = −0.036, *p* = .971).

When controlled for depression symptoms, anxiety symptoms were not associated with subjective ratings (Supplementary Table S9).

### Symptoms and task measures

**Figure 3.**
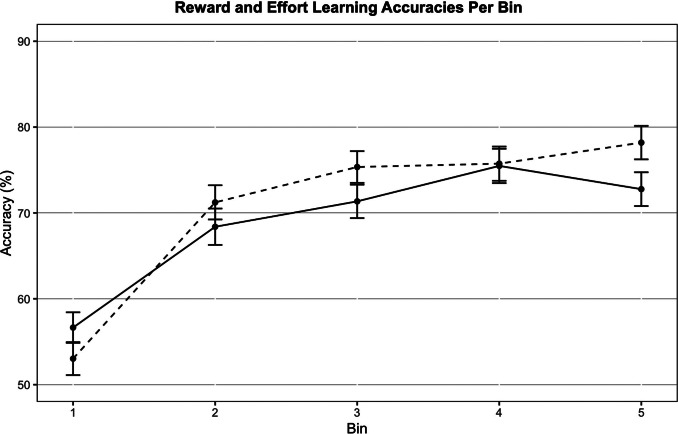
Reward (solid line) and effort learning (dashed line) accuracies across all participants (*N* = 155). Each bin contains five trials. Error bars represent standard errors.

We examined if learning accuracies differed between effort and reward learning blocks, which revealed no significant main effect of learning type (*F*(1,153) = 1.168, *p* = .281) or of block order (*F*(1,153) = 0.032, *p* = .859) on learning accuracy, and no significant interaction (*F*(1,153) = 0.752, *p* = .387). This is also reflected in the reward and effort learning curves, which are similar throughout the block (Figure 3).

**Figure 4.**
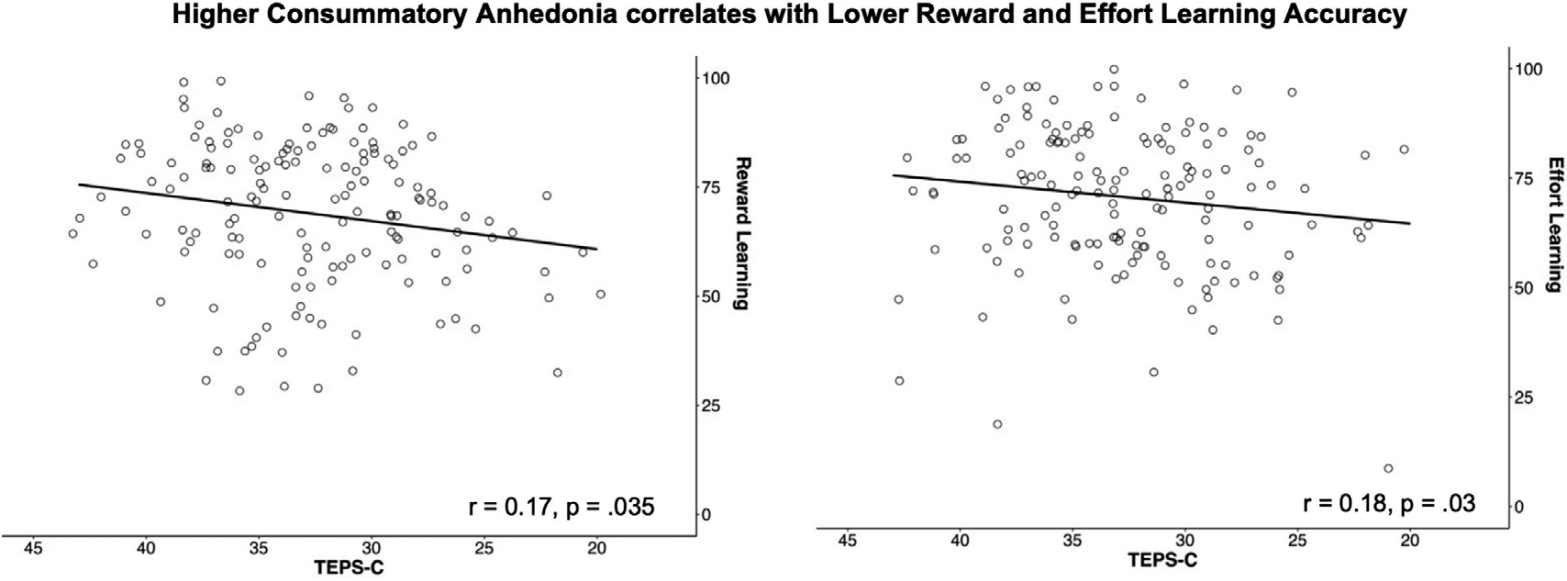
Reward and effort learning accuracy plotted against consummatory anhedonia (higher TEPS-C = lower anhedonia).

We found that, as depression symptoms increased, reward learning accuracy decreased (*r* = −0.17, *p* = .03). No significant relation was found for effort learning accuracy. As consummatory anhedonia increased (TEPS-C), reward learning (*r* = 0.17, *p* = .035) and effort learning accuracies (*r* = 0.18, *p* = .03) decreased (Figure 4). However, none of these findings survived correction for multiple comparisons (Supplementary Tables S3 and S4). There were no significant relationships between learning accuracies and STAI (Supplementary Table S10). Regarding the ability to modulate effort exertion (button presses per sec) as a function of reward, a one-sample *t*-test showed that the difference between effort exerted for high rewards vs low rewards across all participants was significantly greater than 0, indicating that the task was sensitive to effort modulation effects (*t*(144) = 2.459, *p* = .008). Additionally, as anticipatory anhedonia increased (lower TEPS-A), the difference between effort exerted for high vs low rewards was shown to decrease at trend level (*r* = 0.11, *p* = .053), suggesting a poorer ability to modulate effort based on reward value in those with anhedonia symptoms. We found no significant relationship between either depression or anxiety symptoms and effort modulation (see Supplementary Materials).

### Computational modeling

For both reward and effort learning, the best fitting model contained counterfactual learning, with only a learning rate (null and a temperature parameter (*τ*) (Model 2 in Supplementary Table S7). Across all participants, the mean learning rates for reward and effort learning were 0.433 and 0.446, respectively. The mean temperature parameter values for reward and effort learning were 2.049 and 1.541, respectively.

Examining correlations between learning rates, temperature parameters, and symptoms, we found that, as depression scores increased, temperature parameter values for reward learning increased at trend level (*r* = 0.146, *p* = .07). Moreover, as consummatory anhedonia increased, temperature parameter values for reward learning (TEPS-C; *r* = −0.163, *p* = .043) and effort learning (SHAPS; *r* = 0.159, *p* = .049) increased as well (Supplementary Figure S1). However, these results did not survive multiple comparison corrections (Supplementary Tables S5 and S6). No significant relationships were found between the learning rates and depression or anhedonia symptoms. Further, no significant relationships were found for temperature and learning rate parameters with anxiety symptoms (STAI) (Supplementary Table S11).

## Discussion

The main aim of this study was to examine the relationship of depression and anhedonia symptoms to reward and effort learning in young people.

When examining subjective responses, we found anticipatory anhedonia was associated with reduced wanting and liking of reward, consistent with findings from our previous study, also using puppy image rewards (Frey et al., 2023). We also observed that anticipatory anhedonia was associated with reduced willingness to exert effort for reward. This is in line with reports of decreased exertion of grip force for monetary rewards (Cléry-Melin et al., 2011) and reduced choices of high effort/high monetary reward options (Horne et al., 2021) in depression, but extends these previous findings by demonstrating diminished subjective willingness to exert effort for primary rewards in anhedonia. In addition, we showed that lower reward liking was associated with higher consummatory anhedonia and depression symptoms. As consummatory anhedonia was only significantly linked to liking and not wanting of the rewards, this supports the notion that reward sub-processes are subjectively dissociable (Treadway & Zald, 2011).

When examining the task data, we found greater effort exertion for high rewards compared to low rewards, as expected, indicating that the task is sensitive to reward-based effort modulation effects. Moreover, participants’ learning performance was similar for the reward and effort learning blocks, in line with our expectations that using a block design would remove the trade-off between effort and reward learning.

We also found that, as anticipatory anhedonia increased, the difference between the effort exerted for high vs low rewards decreased, indicating that individuals with anhedonia exerted similar effort for high and low rewards. This finding is in line with previous studies showing that depressed individuals fail to exert more effort for higher or more likely rewards (Horne et al., 2021), and with research in schizophrenia reporting that anhedonia is associated with an inefficient effort pattern when trading potential benefits against the associated costs in effort-based decision-making tasks (Fervaha et al., 2013; Ince Guliyev, Guloksuz, & Ucok, 2022; McCarthy, Treadway, Bennett, & Blanchard, 2016; McCarthy, Treadway, & Blanchard, 2015). Our results extend these findings by showing, to the best of our knowledge, for the first time, that anticipatory anhedonia is associated with poorer modulation of effort as a function of reward in young people with depression symptoms.

Further, we demonstrated that, as depression symptoms increased, reward learning accuracy decreased (i.e. the shape that leads to high rewards was chosen less frequently), which is consistent with findings of blunted reward response biases in depression (Esfand et al., 2024; Pechtel, Dutra, Goetz, & Pizzagalli, 2013; Pizzagalli et al., 2008; Pizzagalli, Jahn, & O’Shea, 2005; Vrieze et al., 2013). We also found that, as consummatory anhedonia increased, reward learning accuracies decreased, which is similar to our previous findings (Frey et al., 2023) and in line with studies showing blunted reward response biases with increasing anhedonia (Huys et al., 2013; Kangas et al., 2022; Liu et al., 2016; Vrieze et al., 2013). We extended these findings by also demonstrating, to the best of our knowledge, for the first time, an association between consummatory anhedonia and decreased *effort* learning accuracies. However, as these results did not survive multiple comparison correction, future studies with larger sample sizes are needed to further support these findings.

Using computational modeling, we found that, as consummatory anhedonia increased, temperature parameter values increased for both reward and effort learning. This is consistent with the observation of impaired choice behavior during reward learning in depression (Kunisato et al., 2012; Lloyd et al., 2024; Rupprechter et al., 2018), and a meta-analysis showing more variable choices in anhedonic, depressed, and bipolar individuals in a probabilistic reward task (Huys et al., 2013). However, we have extended these findings by showing, to the best of our knowledge, for the first time, that consummatory anhedonia also correlates with increased temperature parameter values during *effort* learning. This finding could explain the lower reward and effort learning accuracies with increasing consummatory anhedonia observed in this study, as anhedonic individuals underexploit higher-valued choices.

Finally, to assess the specificity of our findings to anhedonia and depression, we also examined whether anxiety symptoms were associated with task performance, subjective ratings, or computational parameters. Previous research has suggested that greater anxiety may lead to avoiding enjoyable activities, resulting in higher anhedonia and depression symptoms (Calafiore, Collins, Bartoszek, & Winer, 2024; Collins et al., 2025; Winer et al., 2017). Our findings, showing that anxiety symptoms were not associated with any of the ratings or parameters, tentatively suggest the absence of an association between anxiety and reward or effort learning when these two aspects are assessed in conjunction. However, this finding is not conclusive and requires further examination.

In terms of study limitations, it should be noted that most of our participants were female, highly educated, Caucasian, and did not have a clinical depression diagnosis. To determine whether our findings generalize beyond this population, future studies should examine the effects of clinical depression and psychiatric medication on performance in the task and recruit a more diverse sample.

In summary, our findings suggest that individuals with anhedonia symptoms may have difficulties appropriately modulating their effortful behavior based on the value of the resulting rewards, and may underexploit options that lead to higher rewards or lower effort requirements. In real life, this may result in the experience of fewer rewarding stimuli and greater effort exertion, which may reduce the hedonic impact of obtained rewards. Therefore, addressing impairments in effort modulation and biases in exploration/exploitation behaviour could be a target for novel interventions for individuals with depression and anhedonia, helping to rebalance effort and reward experiences.

## Supporting information

Sahni et al. supplementary material Sahni et al. supplementary material

## Supplementary material

The supplementary material for this article can be found at http://doi.org/10.1017/S0033291725102523.
