# Supplementary material for: Anhedonia is associated with computational impairments in reward and effort learning in young people with depression symptoms
Source: Psychol Med. 2025 Nov 17;55:e347. doi: 10.1017/S0033291725102523 (PMC13058665; doi:10.1017/S0033291725102523)
Supplement: Sahni et al. supplementary material [file S0033291725102523sup001.docx]

**Supplementary Materials**

**Methods**

*Computational Modelling*

Q-learning models were fit separately to the reward and effort learning data. The Q-values, which represent the predicted outcome value of a given item choice, were initialised at 0.5 for reward learning and at -0.5 for effort learning, as subjects were informed of the learning aim (i.e. maximize reward or minimize effort) in a ‘hint’ page at the start of each block. Q-values were updated on each trial (t) for the selected item (A) as follows:

$$Q_{A}\left( t+1 \right)= Q_{A}\left( t \right)+ \alpha(\rho R\left( t \right)- Q_{A}\left( t \right))$$

where $\alpha$ is the learning rate and $\rho$ is the outcome sensitivity. The outcome value, R(t), was set to 0 and 1 for low and high rewards, respectively, in the reward learning block and to 0 and -1 for low and high effort, respectively, in the effort learning block.

In the models that included counterfactual learning (or double updates), the Q-value of the non-selected item (B) was also updated using the *inverse* of the outcome of the chosen side, R’(t):

$$Q_{B}\left( t+1 \right)= Q_{B}\left( t \right)+ \alpha(\rho R'\left( t \right)- Q_{B}\left( t \right))$$

A SoftMax function was used to determine the probability of participants’ choices (of item A over B) under the model on each trial:

$$P_{A}\left( t \right)=\frac{e^{\frac{Q_{A}\left( t \right)+ {\varphi*c}_{A}\left( t \right)}{\tau}}}{e^{\frac{Q_{A}\left( t \right)+{\varphi*c}_{A}\left( t \right)}{\tau}}+e^{\frac{Q_{B}\left( t \right)+ {\varphi*c}_{B}\left( t \right)}{\tau}}}$$

where τ is the temperature parameter, $c_{A}(t)$ is an indicator variable whose value depends on whether item A was chosen on the previous trial ($c_{A}(t)$ = 1) or not (${c_{A}\left( t \right)= \gamma*c}_{A}\left( t-1 \right)$; $\gamma$ is a decay parameter), and $\varphi$ is a choice bias parameter indicating the likelihood of repeated item choices *independent of their outcomes* (i.e., “sticky choice”; (Schönberg et al., 2007)).

**All models contained a learning rate and temperature parameter, with different combinations of the outcome sensitivity, choice bias and decay parameters added for different models (Supplementary Results). The models not containing these parameters effectively used the above equations with choice bias set to 0, decay set to 1, and/or outcome sensitivity set to 1.**

Each model was fit separately to the reward and effort learning data for each participant by maximising the log likelihood estimate (LLE) of the participant’s choices under the model across all trials within a given block, thus maximising:

$$LLE=ln\left( \prod_{t} P_{i, t} \right)$$

**During model fitting, parameter values were constrained to the following ranges: 0 <** $\boldsymbol{\alpha}$**,** $\boldsymbol{\gamma}$**,** $\boldsymbol{\rho,}\boldsymbol{\varphi}$ **< 1, and 0 < τ < 10. To avoid settling for a local optimum, we employed 10 initialisations using random parameter starting values within the above ranges.**

The relative fit of the different models was compared using Akaike’s Information Criterion (AIC) weights (Wagenmakers & Farrell, 2004). The fit of the best model was compared to chance using *pseudo-R^2^* values (as in (Frank et al., 2007)), comparing the LLE of the learning model to the LLE of the null model, in which $P_{A}\left( t \right)$ is set to 0.5 for each trial. The reward learning data of 14 subjects and the effort learning data of 7 subjects demonstrated a better fit to the null model than to the learning model.

For model validation, data simulations were conducted using the estimated parameter values from the best fitting model. For each participant’s parameter values, 25 simulations were run per block. The accuracy (i.e. whether or not the item with the higher probability of yielding high rewards or low effort was selected) across all trials was determined for each simulation, and the accuracies averaged across the 25 simulations were recorded as the simulated reward and effort learning accuracy of each participant. The simulated accuracy data was then graphically compared to the actual data to assess how well the estimated parameters are able to capture the patterns observed in the actual data. Outputs were examined across all subjects and for only those subjects whose data fit better to the learning than to the null model.

**In addition, the model that generated the simulations was fit back to the simulated data to determine whether the original parameters used to generate the data could be recovered. This yielded 25 learning rate and temperature parameter estimates per participant per block. We calculated the means of the recovered learning rates and temperature parameters per participant for each block, plotted them against the original parameters, and examined the strength of the correlations using the Spearman's method.**

**Results**

*Symptoms and Subjective Ratings*

As reported in the main paper, we used Spearman’s correlations to examine the association between subjective reward ratings and anhedonia and depression symptoms. By applying the Benjamini-Hochberg (BH) method (Benjamini Y, 1995), we corrected for multiple comparisons within each rating type for the anhedonia measures (Table S1), and across the three ratings for the BDI (Table S2).

| **Symptoms** | **Liking** | **Wanting** | **Willingness to  Exert Effort** |
| --- | --- | --- | --- |
| TEPS-A | <.001 | <.001 | .069 |
| TEPS-C | .024 | .126 | .086 |
| SHAPS | .073 | .271 | .477 |
| **Table S1:** *P*-values corrected for multiple comparisons per rating type, using the BH method, for partial Spearman’s correlations controlled for BDI (with anhedonia items removed). | | | |

In addition, we conducted Spearman’s partial correlations, controlling for BDI (with anhedonia items removed), between anhedonia symptoms and learning accuracies (Table S3), as well as full correlations between BDI (full scale) and learning accuracies (Table S4). The below tables report the *p*-values corrected by applying the BH method for multiple comparisons.

| **Symptoms** | **Liking** | **Wanting** | **Willingness to Exert Effort** |
| --- | --- | --- | --- |
| BDI | .048 | .107 | .158 |
| **Table S2: *P*-values** corrected for multiple comparisons using the BH method. | | | |

| **Symptoms** | **Reward Learning** | **Effort Learning** |
| --- | --- | --- |
| TEPS-A | .925 | .537 |
| TEPS-C | .111 | .081 |
| SHAPS | .925 | .086 |
| \| **Symptoms** \| **Reward Learning** \| **Effort Learning** \| \| --- \| --- \| --- \| \| BDI \| .060 \| .150 \| \| **Table S4:** *P*-values of BDI correlations with reward and effort learning accuracies corrected for multiple comparisons using the BH method. \| \| \|   **Table S3:** *P*-values corrected for multiple comparisons for reward and effort learning accuracies, using the BH method, for partial Spearman’s correlations controlled for BDI (with anhedonia items removed). | | |

*Symptoms and Parameters*

Higher consummatory anhedonia levels were significantly associated with higher reward learning temperature parameters (higher $\tau$ with lower TEPS-C scores; r = -0.163, p = .043) and with higher effort learning temperature parameters (higher $\tau$ with higher SHAPS scores; r = 0.159, p = .049). These results are graphically represented in Figure S1**.**


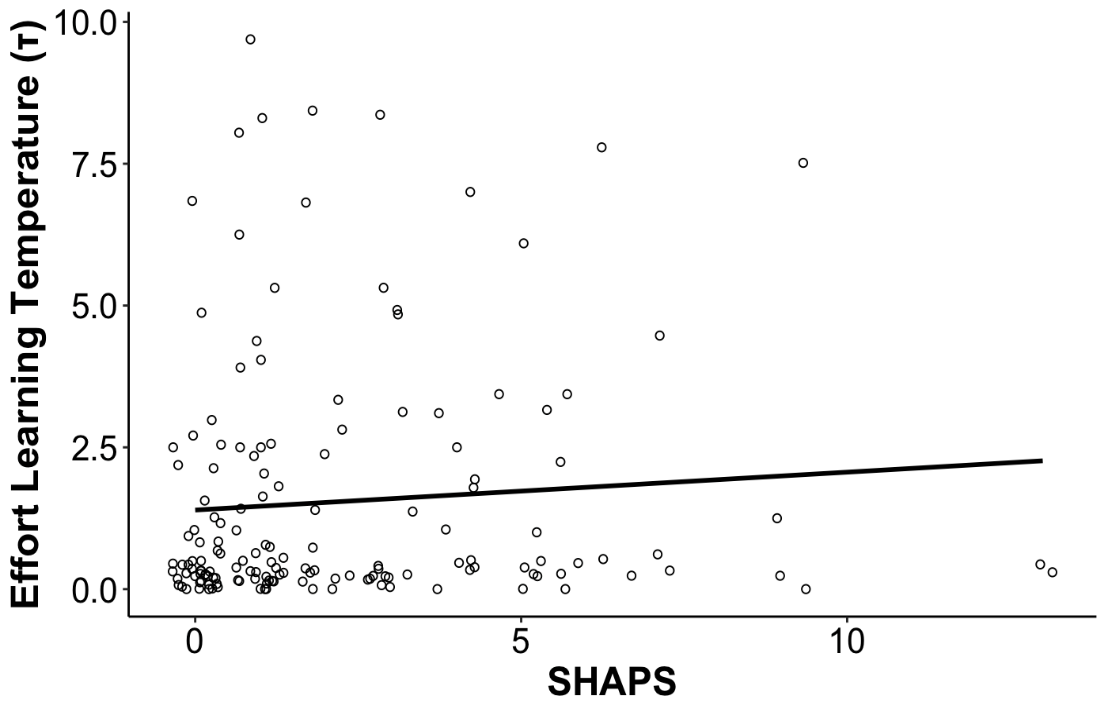

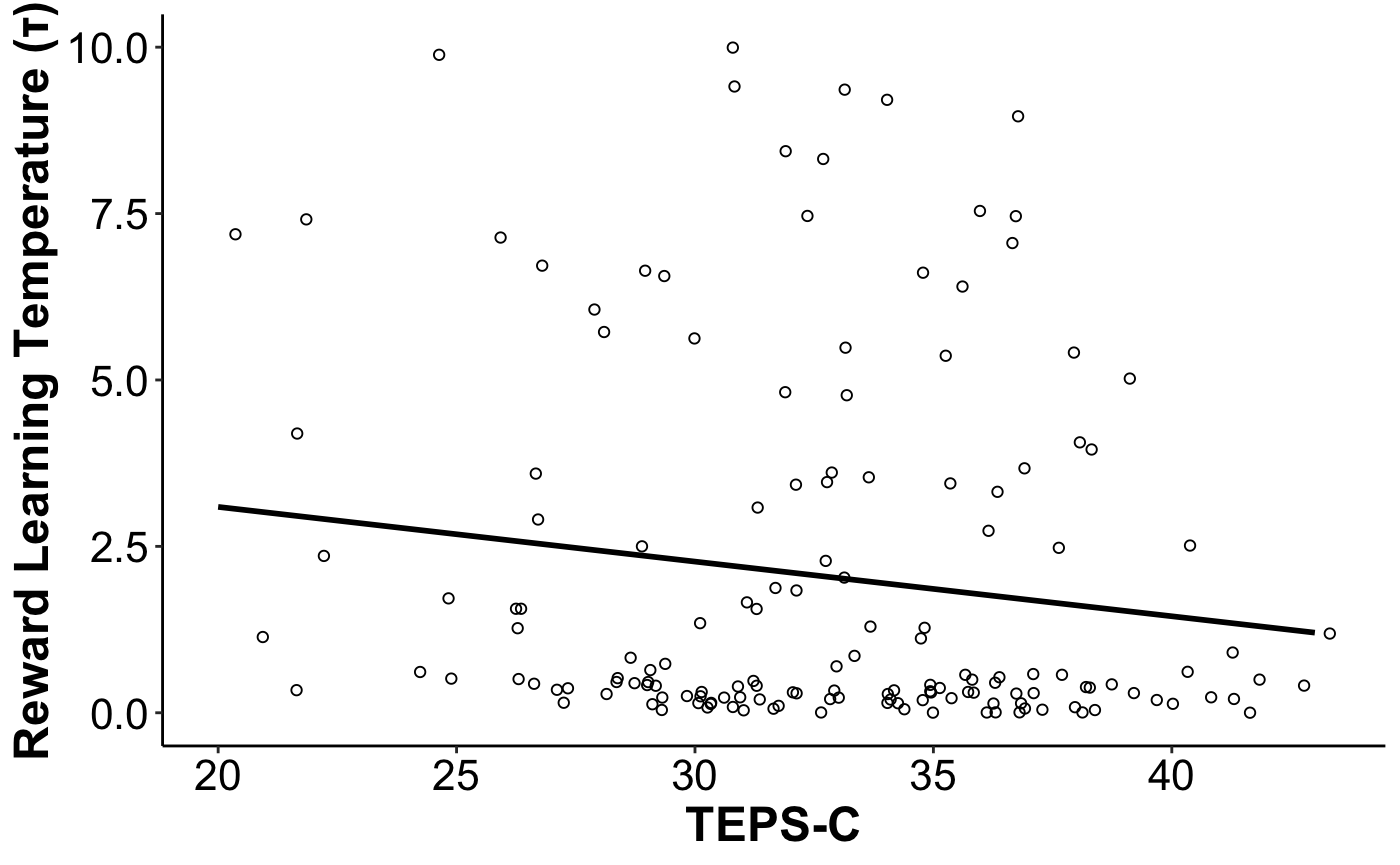


**Figure S1:** Reward and effort Learning temperature ($\tau$) correlations with consummatory anhedonia. In both blocks, higher consummatory anhedonia was associated with higher temperature parameter values.

r = -0.163,
p = .043

r = 0.159, p = .049

Below the corrected *p*-values using the BH method are reported for the correlations of temperature parameters with anhedonia (Table S5) and depression (Table S6) symptoms. No significant effects survived correction for multiple comparisons.

| **Symptoms** | **Reward** $\boldsymbol{\tau}$ | **Effort** $\boldsymbol{\tau}$ |
| --- | --- | --- |
| TEPS-A | .503 | .645 |
| TEPS-C | .129 | .128 |
| SHAPS | .503 | .128 |
| **Table S5:** *P*-values corrected for multiple comparisons for reward and effort learning temperature parameters, using the BH method, for partial Spearman’s correlations controlled for BDI (anhedonia items removed). | | |

| **Symptoms** | **Reward** $\boldsymbol{\tau}$ | **Effort** $\boldsymbol{\tau}$ |
| --- | --- | --- |
| BDI | .140 | .618 |
| **Table S6:** *P*-values for BDI correlations with reward and effort learning temperature ($\tau$) parameters, corrected for multiple comparisons using the BH method. | | |

*Model Selection*

Akaike’s Information Criterion (AIC) weights indicated that the model using counterfactual updating and containing only a learning rate and a temperature parameter (Model 2, Table S7) provided the best fit for both the reward and effort learning data. AIC weights for all models are shown in Table S7.

| **Model** | **α** | $\boldsymbol{\gamma}$ | $\boldsymbol{\varphi}$ | **ρ** | $\boldsymbol{\tau}$ | **Update** | **AIC weights reward** | **AIC weights effort** |
| --- | --- | --- | --- | --- | --- | --- | --- | --- |
| 1 | X |  |  |  | X | factual | 0.245 | 0.267 |
| **2** | **X** |  |  |  | **X** | **counterfactual** | **0.509** | **0.494** |
| 3 | X |  |  | X | X | factual | 0.016 | 0.005 |
| 4 | X |  |  | X | X | counterfactual | 0.098 | 0.075 |
| 5 | X | X |  |  | X | factual | 0.032 | 0.029 |
| 6 | X | X |  |  | X | counterfactual | 0.065 | 0.107 |
| 7 | X |  | X |  | X | factual | 0.009 | 0.008 |
| 8 | X |  | X |  | X | counterfactual | 0.01 | 0.009 |
| 9 | X | X | X |  | X | factual | 0.005 | 0.002 |
| 10 | X | X | X |  | X | counterfactual | 0.01 | 0.004 |
| **Table S7:** Akaike’s Information Criterion (AIC) weights indicate the relative fit of different models, with higher weights representing a better fit. Model 2 shows the best fit to both reward and effort learning data. ‘Update’ refers to whether only factual outcomes, or factual and counterfactual outcomes, were used to update the Q-values. | | | | | | | | |

*Model Validation*

Graphically, comparing the accuracy across trials between the actual and simulated data revealed that, although the overall accuracies were slightly underestimated in the simulations, the relative accuracy pattern of the simulated data resembled that of the real data in the whole sample (Figure S2) and after the removal of participants whose data fit the null model better than the learning model (Figure S3). Additionally, the *pseudo-R^2^* values for the whole sample indicated that, in both reward (*pseudo-R^2^* = 0.28) and effort learning (*pseudo-R^2^* = 0.32) blocks, the model provided a relatively good fit for the data.

**To assess if symptoms were associated with how well Model 2 fit the choice data, we correlated *pseudo-R^2^* values with depression and anhedonia symptoms. We found that lower TEPS-C scores were associated with lower *pseudo-R^2^* values for reward learning (*r* = 0.183, *p* = .023), suggesting a poorer fit of Model 2 for reward learning in those with higher consummatory anhedonia (Table S8). This correlation did not survive correction for multiple comparisons by applying the BH method (*p* = .092).**

| **Symptoms** | **Reward *Pseudo-R^2^*** | **Effort *Pseudo-R^2^*** |
| --- | --- | --- |
| BDI* | *r* = -0.128, p = .112; corrected *p* = .224 | *r* = 0.006, p = .939; corrected *p* = .939 |
| TEPS-A | *r* = 0.081, p = .318; corrected *p* = .424 | *r* = 0.035, p = .67; corrected *p* = .893 |
| TEPS-C | *r* = 0.183, p = .023; corrected *p* = .092 | *r* = 0.129, p = .112; corrected *p* = .238 |
| SHAPS | *r* = 0.051, p = .531; corrected *p* = .531 | *r* = -0.127, p = .119; corrected *p* = .238 |
| **Table S8:** Spearman's correlation of symptoms with reward and effort learning block's *Pseudo-R^2^* from Model 2. Anhedonia symptoms were controlled for BDI (anhedonia items removed).  BDI* = full BDI scale (no items removed). | | |


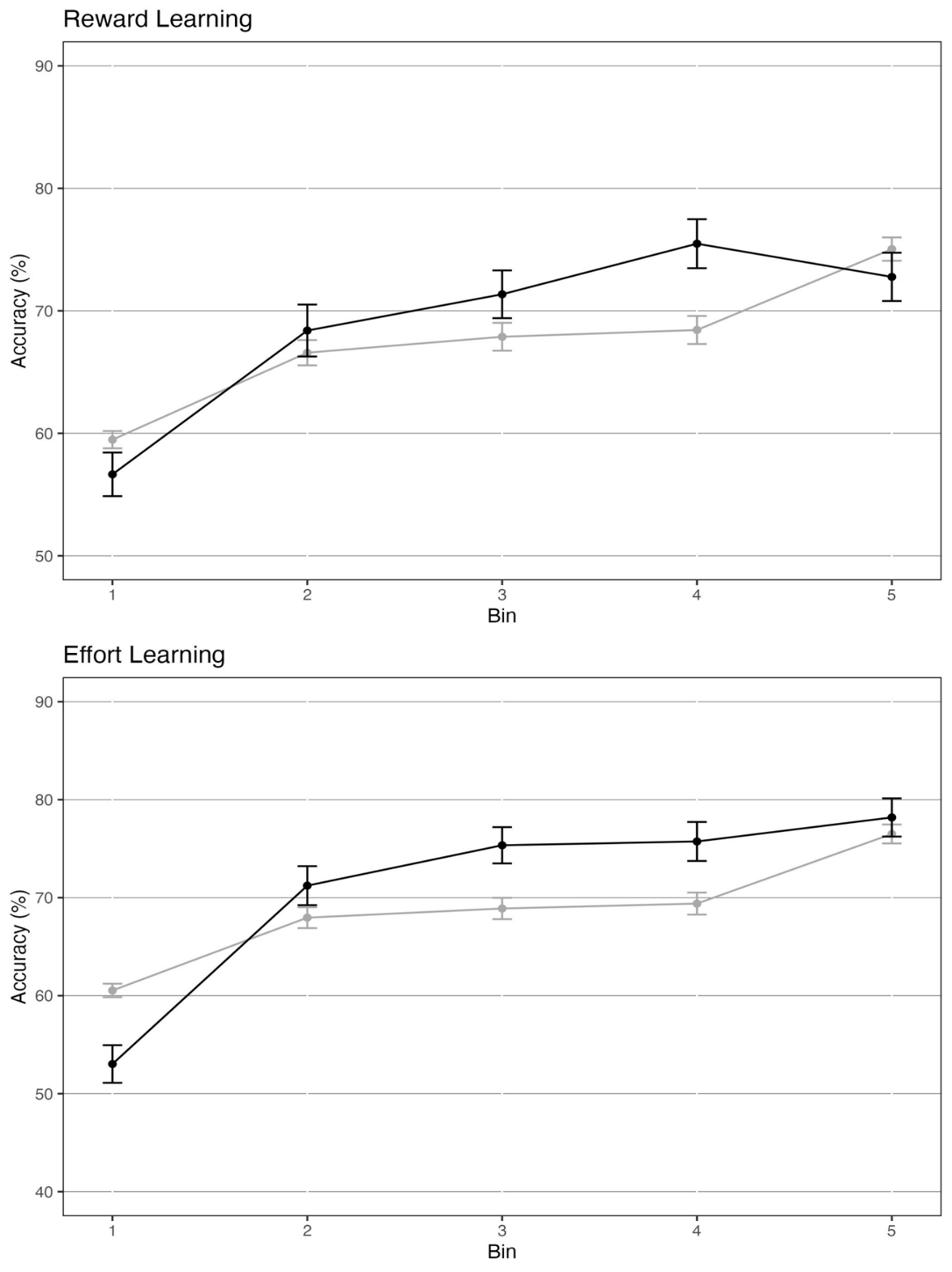


**Figure S2:** Reward and effort learning accuracies across all participants (N = 155) based on actual data (black) and simulated data using parameter estimates from the best fitting model (grey). Each bin contains 5 trials.


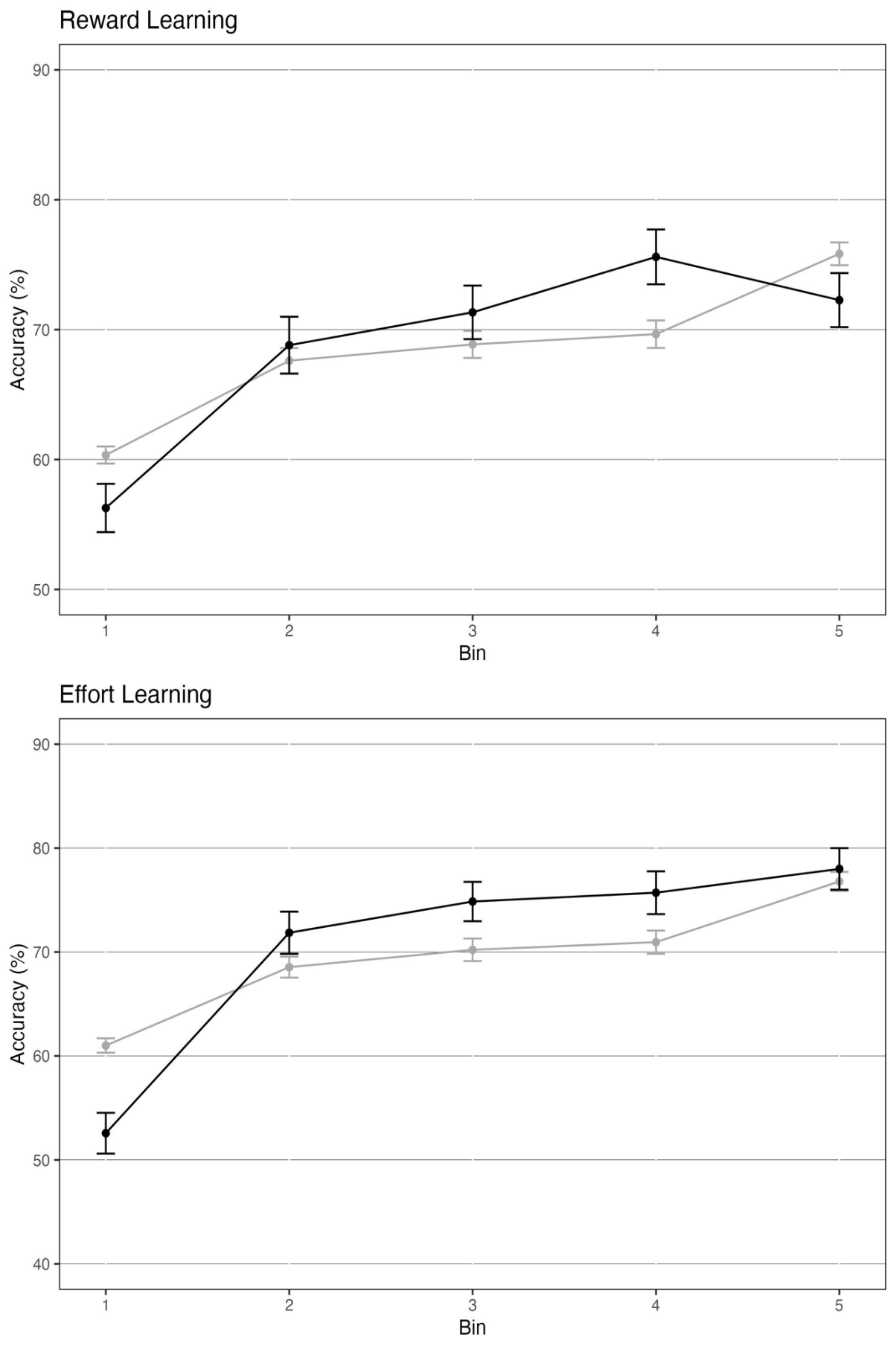


**Figure S3:** Reward and effort learning accuracies after removing participants whose data fit the null model better than the learning model (reward learning n removed = 14; effort learning n removed = 7) based on actual data (black) and simulated data using parameter estimates from the best fitting model (grey). Each bin contains 5 trials.

Lastly, we observed satisfactory parameter recovery, with moderate to strong correlations between most original and recovered parameter values (Figures S4 and S5).


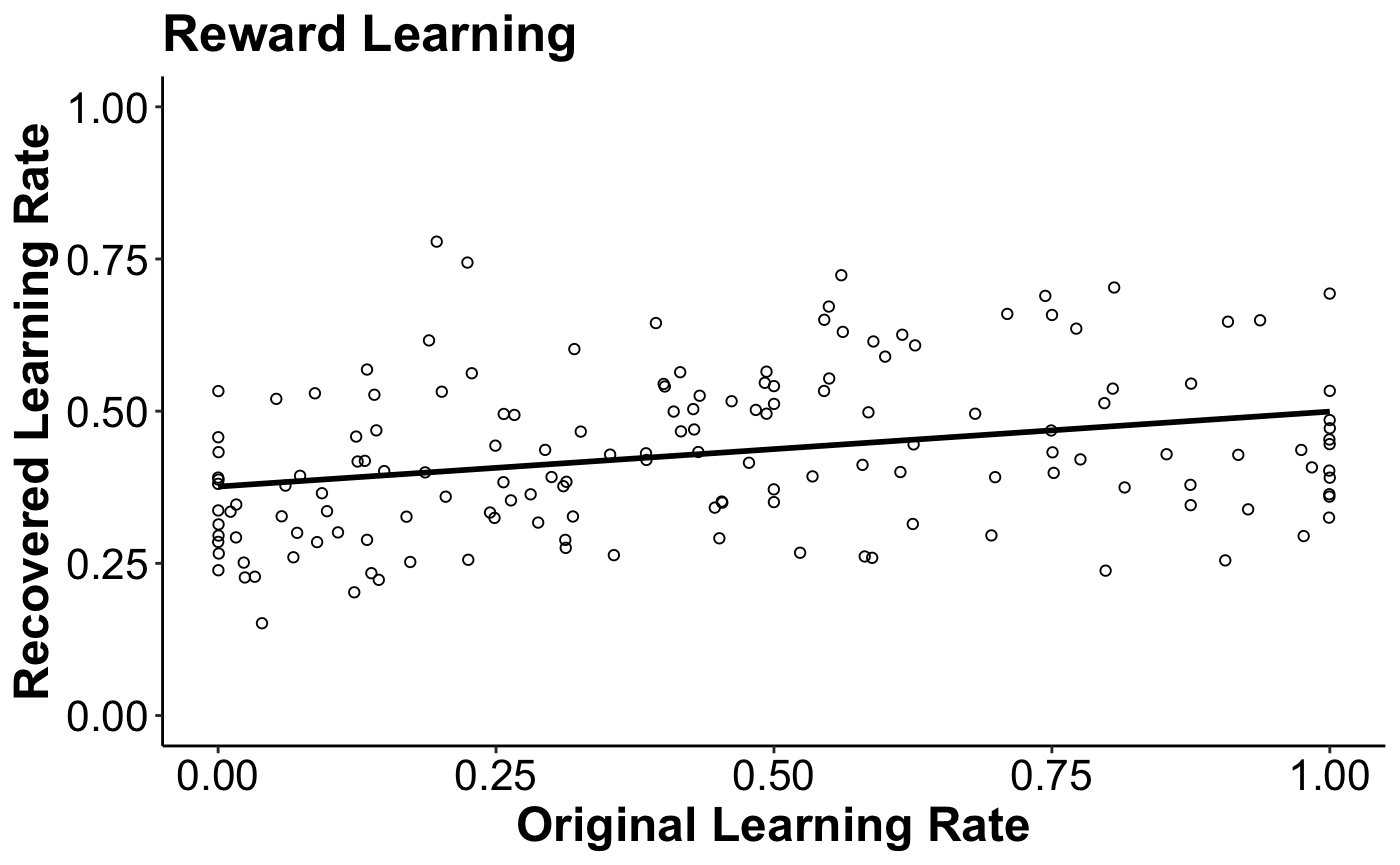

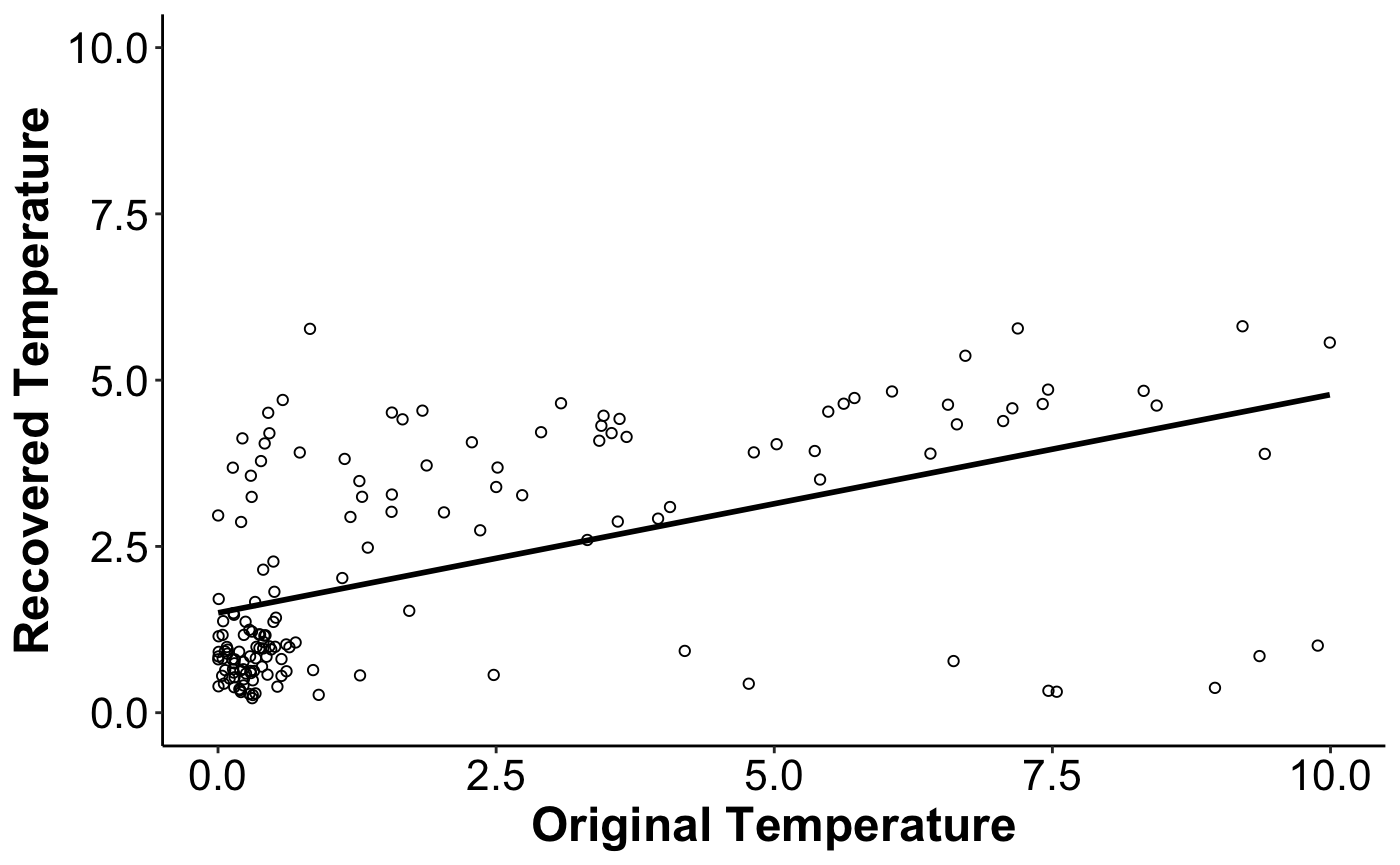


r = 0.34, p<.001

r = 0.55, p<.001

**Figure S4:** Correlations between recovered and original parameters for reward learning.


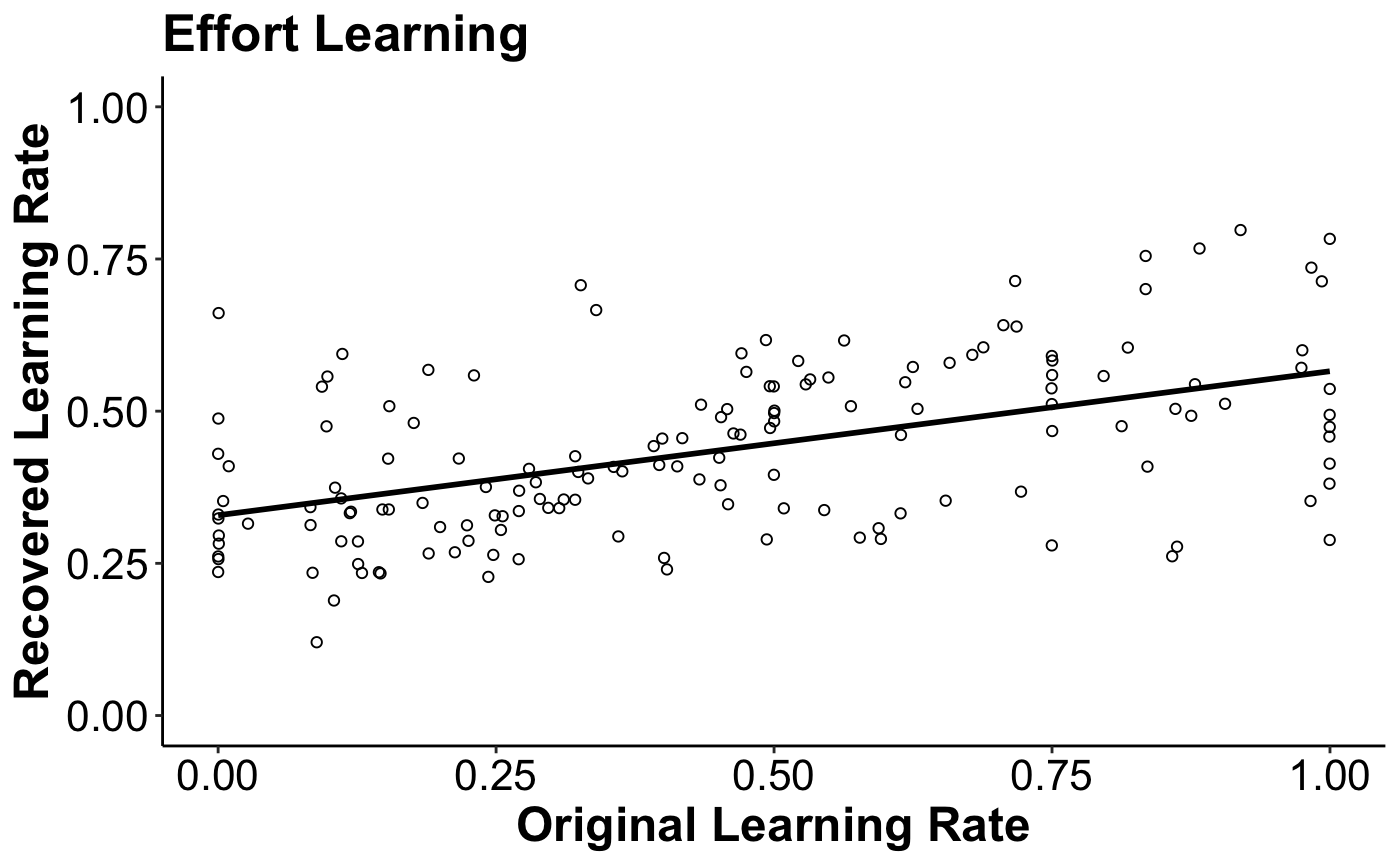

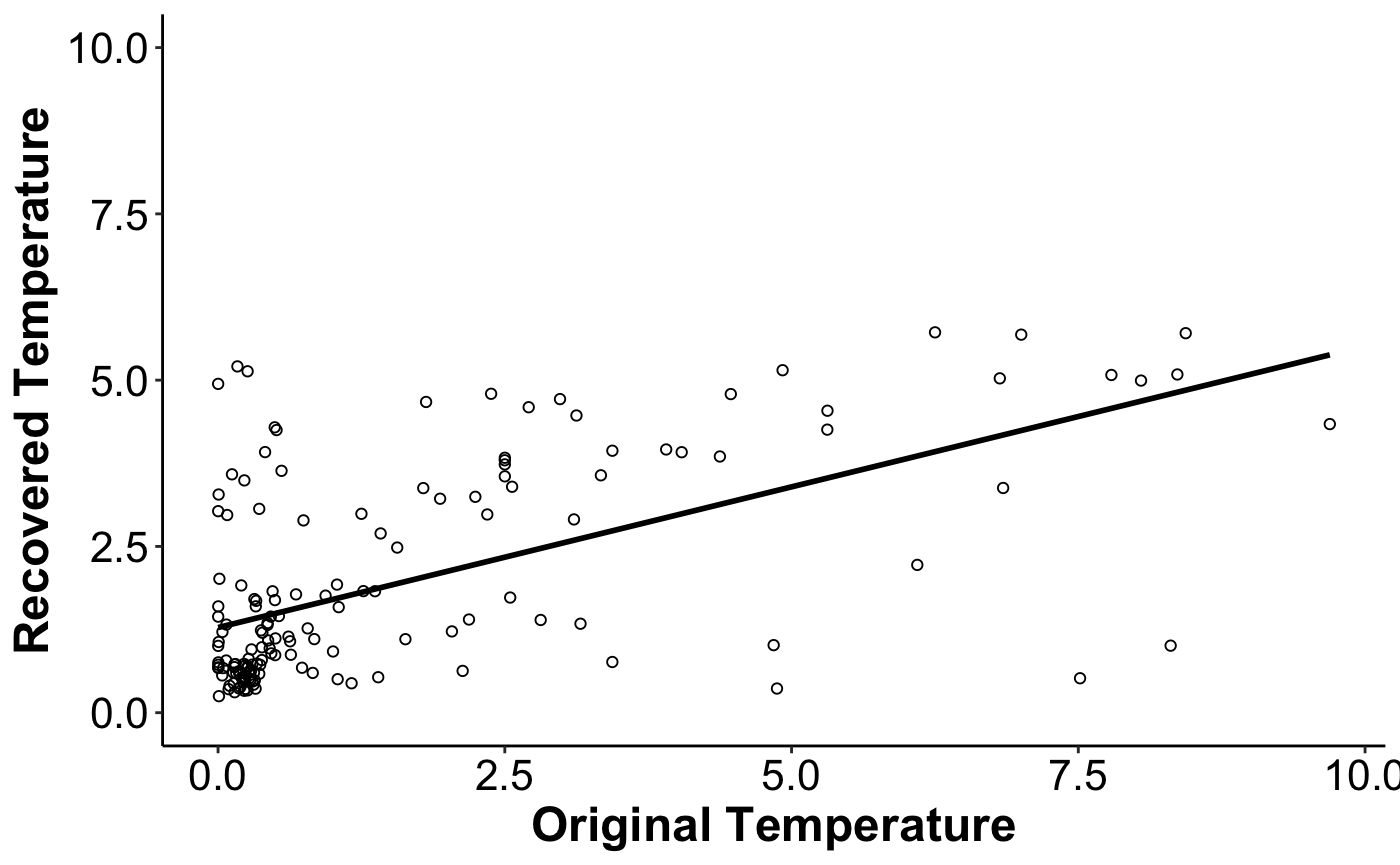


r = 0.51, p<.001

r = 0.53, p<.001

**Figure S5:** Correlations between recovered and original parameters for effort learning.

*Anxiety Symptoms*

To assess the specificity of our findings to depression and anhedonia, we examined whether or not task measures were also related to anxiety symptoms (as measured by the State Trait Anxiety Inventory (STAI; Bieling et al., 1998)) using partial Spearman’s correlations, controlled for depression (BDI, full scale). We also corrected for multiple comparisons by applying the BH method (Benjamini Y, 1995).

We found no significant relationships of anxiety symptoms with subjective ratings (Table S9), learning accuracies (Table S10) or computational parameters (Table S11).

|  | **Liking** | **Wanting** | **Willingness to Exert Effort** |
| --- | --- | --- | --- |
| Correlation coefficients with uncorrected *p*-values | *r* = -0.057  *p* = .484 | *r* = -0.071 *p* = .382 | *r* = -0.098  *p* = .226 |
| Corrected *p*-values | *p* = .484 | *p* = .484 | *p* = .484 |
| **Table S9:** Spearman’s correlations of STAI scores with liking, wanting and willingness to exert effort, controlling for BDI. The *p*-values were corrected by applying the BH-method. | | | |

|  | **Reward Learning** | **Effort Learning** |
| --- | --- | --- |
| Correlation coefficients with uncorrected *p*-values | *r* = 0.068  *p* = .404 | *r* = 0.009  *p* = .914 |
| Corrected *p*-values | *p* = .808 | *p* = .914 |
| **Table S10:** STAI correlations with reward and effort learning accuracies, controlled for BDI. The *p*-values were corrected by applying the BH-method. | | |

|  | **Reward α** | **Reward** $\boldsymbol{\tau}$ | **Effort α** | **Effort** $\boldsymbol{\tau}$ |
| --- | --- | --- | --- | --- |
| Correlation coefficients with uncorrected *p*-values | *r* = -0.068  *p* = .400 | *r* = -0.053  *p* = .515 | *r* = 0.017 *p* = .836 | *r* = -0.046  *p* = .567 |
| Corrected *p*-values | *p* = .756 | *p* = .756 | *p* = .836 | *p* = .756 |
| **Table S11:** STAI correlations with reward and effort learning temperature ($\tau$) and learning rate (α) parameters, controlled for BDI. The *p*-values were corrected by applying the BH-method. | | | | |

**References**

Benjamini Y, H. Y. (1995). Controlling the false discovery rate: a practical and powerful approach to multiple hypothesis testing. . *J R Stat Soc B 57*, 289–300.

Bieling, P. J., Antony, M. M., & Swinson, R. P. (1998). The State--Trait Anxiety Inventory, Trait version: structure and content re-examined. *Behaviour Research and Therapy*, *36*(7), 777-788. <https://doi.org/https://doi.org/10.1016/S0005-7967(98)00023-0>

Frank, M. J., Moustafa, A. A., Haughey, H. M., Curran, T., & Hutchison, K. E. (2007). Genetic triple dissociation reveals multiple roles for dopamine in reinforcement learning. *Proceedings of the National Academy of Sciences*, *104*(41), 16311-16316. <https://doi.org/doi:10.1073/pnas.0706111104>

Schönberg, T., Daw, N. D., Joel, D., & O'Doherty, J. P. (2007). Reinforcement learning signals in the human striatum distinguish learners from nonlearners during reward-based decision making. *J Neurosci*, *27*(47), 12860-12867. <https://doi.org/10.1523/jneurosci.2496-07.2007>

Wagenmakers, E.-J., & Farrell, S. (2004). AIC model selection using Akaike weights. *Psychonomic Bulletin & Review*, *11*(1), 192-196. <https://doi.org/10.3758/BF03206482>
